# A SWOT analysis of nano co-crystals in drug delivery: present outlook and future perspectives

**DOI:** 10.1039/d3ra00161j

**Published:** 2023-03-07

**Authors:** Bwalya A. Witika, Yahya E. Choonara, Patrick H. Demana

**Affiliations:** a Department of Pharmaceutical Sciences, School of Pharmacy, Sefako Makgatho Health Sciences University Pretoria 0208 South Africa bwalya.witika@smu.ac.za; b Wits Advanced Drug Delivery Platform Research Unit, Department of Pharmacy and Pharmacology, School of Therapeutic Science, Faculty of Health Sciences University of the Witwatersrand 7 York Road, Parktown Johannesburg 2193 South Africa

## Abstract

The formulation of poorly soluble drugs is an intractable challenge in the field of drug design, development and delivery. This is particularly problematic for molecules that exhibit poor solubility in both organic and aqueous media. Usually, this is difficult to resolve using conventional formulation strategies and has resulted in many potential drug candidates not progressing beyond early stage development. Furthermore, some drug candidates are abandoned due to toxicity or have an undesirable biopharmaceutical profile. In many instances drug candidates do not exhibit desirable processing characteristics to be manufactured at scale. Nanocrystals and co-crystals, are progressive approaches in crystal engineering that can solve some of these limitations. While these techniques are relatively facile, they also require optimisation. Combining crystallography with nanoscience can yield nano co-crystals that feature the benefits of both fields, resulting in additive or synergistic effects to drug discovery and development. Nano co-crystals as drug delivery systems can potentially improve drug bioavailability and reduce the side-effects and pill burden of many drug candidates that require chronic dosing as part of treatment regimens. In addition, nano co-crystals are carrier-free colloidal drug delivery systems with particle sizes ranging between 100 and 1000 nm comprising a drug molecule, a co-former and a viable drug delivery strategy for poorly soluble drugs. They are simple to prepare and have broad applicability. In this article, the strengths, weaknesses, opportunities and threats to the use of nano co-crystals are reviewed and a concise incursion into the salient aspects of nano co-crystals is undertaken.

## Abstract 2

The formulation of poorly soluble drugs is an intractable challenge in the field of drug design, development and delivery.

## Graphical abstract

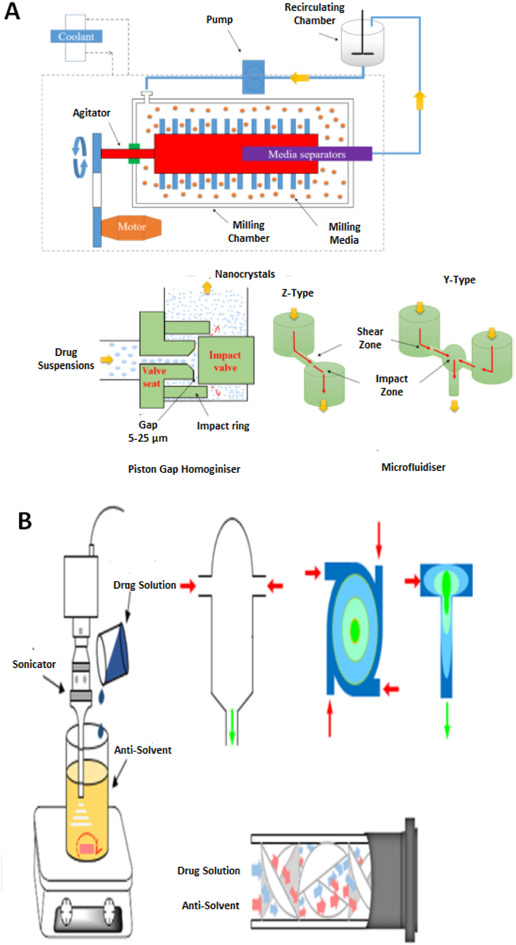

## 1 Introduction

Recently, there have been notable advancements in combinatorial chemistry, high throughput screening and *in silico* drug candidate discovery resulting in many potential drug candidates with excellent target receptor binding. However, many of these candidates have properties such as a large molecular weight and high log *P* values that hinder their formulation into a final pharmaceutical drug product.^1^ Such molecules have inherently low aqueous solubility as a major limitation. Two relatively similar drug synthesis techniques, *i.e.* crystal engineering and nanocrystallisation have been proposed to circumvent the poor aqueous solubility of many drug candidates.^2–6^ Crystal engineering is the use and manipulation of non-covalent interactions between molecular or ionic components for the rational design of solid-state structures that may have different and exciting biological, pharmaceutical, electrical, magnetic, and optical properties compared to the parent molecules.^2^ It is apparent that the directionality, specificity and predictability of intermolecular hydrogen bonds can be exploited to assemble supramolecular structures that, at a minimum, can control or influence dimensionality.^2,7^ It further utilises the understanding of intermolecular interactions in the context of crystal packing to design new solid materials with desired physicochemical properties such as solubility, permeability, stability, hygroscopicity, wettability, hydration, colour, compaction, tableting and bioavailability.^8–10^ Molecules exist as distinct solid forms that can broadly be defined as polymorphs, co-crystals, salts, solvates and amorphous solids.^11^ Active pharmaceutical ingredients (API) are frequently administered as solid-state polycrystalline materials formulated into an appropriate dosage form.

Solid dosage forms are a convenient for API storage. API can exist in a variety of solid-state forms, in which each form may display unique physicochemical properties such as differences in hygroscopicity, wettability, hydration, colour, compaction, tableting, permeability, stability, and bioavailability morphology, melting point and solubility. However, some potentially useful compounds with highly desirable molecular pharmacological properties may never realise their therapeutic potential due to poor solubility and bioavailability, undesirable processing characteristics and a short shelf-life stability.^12,13^

Co-crystals are defined as single-phase crystalline materials constituting two or more different molecular or ionic compounds combined in molar ratios.^14^ Different types of molecular bonds can be utilised to construct co-crystals and includes π–π stacking, hydrogen bonds, van der Waal's forces and ionic interactions.^15–18^ Co-crystals tend to be more thermodynamically stable than the crystalline solids of pristine compounds. For pharmaceutical applications, they are highly promising to improve the physicochemical properties of an API.^19^ When pairing the API with a co-former (*i.e.* a molecule selected to co-crystallise with the API), there is potential for improving the biopharmaceutical and physicochemical properties of the API such as the dissolution kinetics, bioavailability and/or pharmaceutical stability of the API.^17,19–21^

Aside from engineering co-crystals, nano-crystallisation is another progressive approach to improve the limitations of pristine molecules.^22^ It is well-reported that particle size reduction results in an increase in surface area and subsequently a significant enhancement of surface-active phenomena such as dissolution rates and bioavailability in accordance with the Noyes–Whitney relationship.^23^

Pharmaceutical nanocrystals are nanoscale, heterogeneous aqueous dispersions of insoluble drug particles stabilised by surfactants and/or polymers.^1,22,24^ Generally, nanocrystals are considered to be in the range of sub-micron dimension.^25–27^ A summary of the properties of nanocrystals and co-crystals is depicted in Table 1.

**Table 1:** Summary of the general features of pure drug nanocrystals and co-crystals for drug delivery^28,29^

Pure nanocrystals	Co-crystals
Particle size < 1 μm	Enhanced dissolution rate
100% drug (no carrier)	Enhanced intrinsic solubility
Increased dissolution rate	Enhanced melting point
Increased saturation solubility	Enhanced hygroscopicity
Increased adhesiveness to surface/cell membranes	Enhanced compressibility
Increased bioavailability of the drug	Enhanced bulk density
Possible nanotoxicity and side effects	Enhanced friability
Generally needed to be stabilized by surface active agent	
Long-term stability concerns	

Therefore, merging co-crystallisation with nano-sizing of a drug candidate may be desirable as the resultant nano co-crystals (NCCs) can exhibit the combined improved physicochemical and biopharmaceutical properties when compared to the parent molecule(s).

In this paper, the strengths and weaknesses of this relatively new combinatorial strategy (*i.e.* NCCs) is assimilated and a concise incursion into the various opportunities and threats is discussed. This SWOT analysis and future direction of NCCs in drug delivery is also provided.

## 2 Strengths

Nano co-crystals (NCCs) have several strengths as they combine the advantages of co-crystals with nano-sizing. A multitude of synthesis techniques exist to improve the physicochemical properties, flexibility of route of administration and the potential for secondary processing to obtain superior outcomes from new drug candidates.

### 2.1 Critical processing parameters (CPP)

Critical processing parameters (CPPs) are defined as procedural constraints whose variability has an impact on the critical quality attributes (CQAs) and, therefore, CPPs should be monitored or controlled to ensure that the synthesis procedure produces the desired API quality.^30^ Optimization of the CQAs further leads to the development of drug candidates within the ambit of the desirable quality target product profile (QTPP). Individual CPPs do not essentially impact all CQAs. In many instances, multiple CPPs have an impact on one CQA.^31,32^ NCCs have can be manufactured using a variety of synthesis techniques that allow for the variation of CPPs to attain a final product with the desired QTPPs.

#### 2.1.1 Various methods to synthesize NCCs

**Fig. 1:**
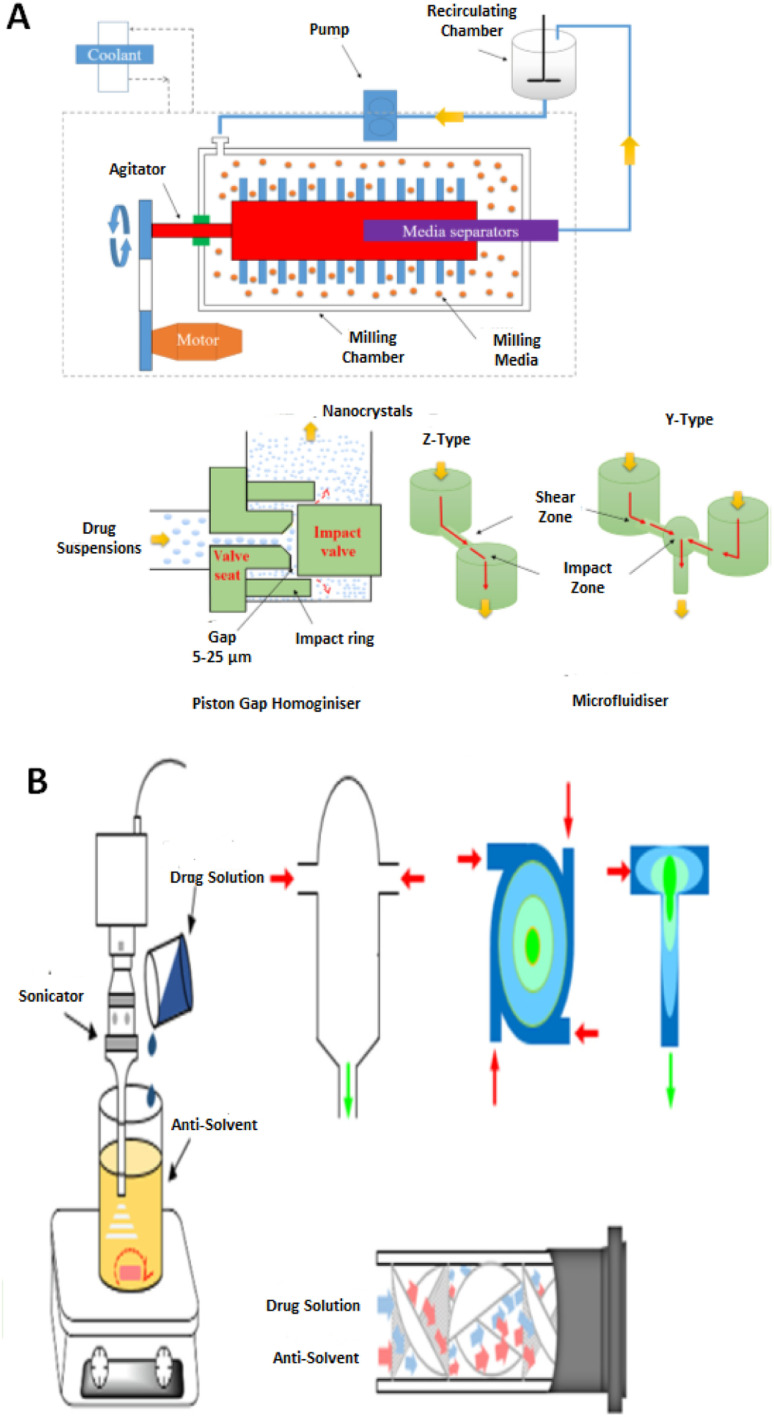
An illustration of the top-down (A) and bottom-up (B) strategies for drug nano co-crystal manufacturing in the presence of stabilisers. Adapted from ref. 130 with permission from Elsevier B.V Amsterdam in accordance with Creative Commons 4.0 (http://creativecommons.org/licenses/by-nc-nd/4.0/).

A major advantage of NCCs is that, much like co-crystals and nanocrystals, they can be synthesized using two approaches *viz.*, a top-down technique that utilizes shear forces to reduce the particle size (mm μm^−1^ to nm)^33,34^ or a bottom-up approach that involves nucleation and growth of individual monomers that are halted, electrostatically and/or sterically at the nanoscale using stabilisers such as surfactants or polymers.^35,36^ A summary of the general design principle for each technique is provided in Fig. 1.

The ability to synthesize NCCs using various techniques allow for flexibility in the manufacturing process to produce NCCs of the desired pharmaceutical attributes.

##### 2.1.1.1 Top-down techniques

High-energy mechanical forces are involved in top-down approaches to produce NCCs. These include media milling (MM) *viz.*, NanoCrystals® or HPH, IDD-P®, DissoCubes® and Nanopure® to comminute larger crystals to the nanometer scale.^34,37^ Top-down approaches are universal for preparing crystalline nanoparticles^38^ and are flexible at production scale.^39^ The process has been widely adopted to prepare nanocrystals on a commercial scale.

For example, the HPH approach has been successfully utilized to develop baicalein-nicotinamide (BE-NCT) NCCs.^40^ BE-NCT NCCs were prepared using HPH with poloxamer 188 as a stabiliser in a formulation containing 2% w/w BE-NCT co-crystals and 0.4% w/w poloxamer 188. The system was initially homogenised at 15 000 rpm for 10 min using a lab-scale high shear dispersing emulsifier after which HPH using 900 bar was applied for 20 homogenisation cycles to produce a suspension of BE-NCT NCCs.^41^

Top-down techniques have also been used with considerable success for the diuretic API furosemide to produce NCCs with acetamide, urea, caffeine, and nicotinamide as the selected co-formers. Similarly, carbamazepine and indomethacin were separately prepared as NCCs using saccharin as the co-former.^42^ The initial step involved the synthesis of micronized co-crystals of each using liquid assisted grinding (LAG) with methanol, acetonitrile, acetone or using a slurry technique.^42^ The resultant co-crystals were dispersed into a solution containing 0.5% w/v HPMC and 0.02% w/v sodium dodecyl sulphate (SDS) in distilled water and wet-milled with zirconia beads. Wet milling was conducted thrice at ∼ 2000 rpm for 2 min and 500 rpm for 2 min cycles. The milling chamber was maintained at −10 °C during the process. The resulting suspension and zirconia bead mixture was transferred to a centrifuge filter-mesh chamber to separate and collect the suspensions at 400 rpm for 1 min.^42^ Stability and dissolutions assessments were conducted on the NCCs. In the stability study, the prepared furosemide–caffeine, carbamazepine–saccharin, and indomethacin–saccharin NCCs exhibited physical and chemical stability for one month at 5 and 25 °C.

NCCs of diclofenac-proline was also synthesized by a top-down approach using wet milling and neat grinding.^43^ The first step was to grow diclofenac-proline co-crystals (DPC) using LAG. To nano-size these, wet milling was used in which 500 mg of DPC was placed in a mortar and mixed with 5% polyvinylpyrrolidone (PVP) and 5% of Tween® 80 in ethanol while milling. The clear mixture was placed in a glass beaker. The solution was then sonicated for 5, 10, 15, 20, and 30 min at 30 °C before evaluation. For the neat grinding procedure, 1 g DPC was ground for ∼8 h while adding ethanol. At each hour, a sample of NCCs was dissolved in sodium lauryl sulphate (SLS) 0.1% w/w. The NCCs exhibited significant improvements in dissolution rates when compared to diclofenac coarse powders.^43^

De Smet and co-workers produced NCCs for the anti-fungal API itraconazole using wet milling. This was achieved by mixing 250 mg itraconazole with Tween® 80 and dispersing both in 5 ml demineralised water in a 20 ml vial.^44^ Different concentrations of dicarboxylic acid co-formers *viz.*, maleic, adipic, glutaric and succinic acid were dissolved in the suspension. Milling beads made from zirconium oxide with a weight and diameter of 30 g of 500 μm diameter, respectively were added to the suspension. Subsequently, the milling chambers were placed on a roller-mill and milling was accomplished at 150 rpm for 60 h after which the NCCs were separated from the milling media by sieving.^44^ The NCCs hereby formed were compared to commercially available market comparator. The NCCs yielded provided faster release from the solid dosage form compared to market comparator.^44^

Huang *et al.*, successfully manufactured itraconazole and indomethacin NCCs.^45^ Itraconazole and indomethacin macro co-crystals were initially manufactured using solvent evaporation utilizing succinic acid and fumaric acid as CCF. It was observed that the resulting NCCs showed remarkable increased kinetic solubility and dissolution rates compared to nanocrystals and co-crystals when analysed using *in situ* kinetic solubility studies.^45^ The solubility of the novel itraconazole-succinic acid NCCs was further assessed for solubility in the presence of different polymers.^46^ The polymer that yielded the best NCC solubility enhancement was then utilized as a binding agent to formulate a solid oral dosage form using itraconazole-succinic acid NCC.^47^ The NCC loaded multiparticulates were then used to assess the influence of different drying processes, types of substrates, and drug loading on drug dissolution under non-sink conditions.^47^ Furthermore, a comparative assessment was carried out with the optimized dried dosage forms commercial product sugar beads for *in vitro* dissolution performance.^47^ The comparison with market comparator indicated NCC downstreaming using bead layering might be an alternative or even superior choice to previous approaches for the development of novel solid oral itraconazole formulations.^47^

A multidrug NCC was manufactured in attempt to develop a treatment modality to co-deliver lamivudine and zidovudine for the management of HIV.^48^ The initial step involved the manufacture of micronized co-crystals using solvent evaporation with ethanol and water. The optimized NCC had residue contamination from the milling media. However, the NCC exhibited improved cell viability when compared to the individual API.^48^

NCC of myricetin and nicotinamide have been manufactured using a neat grinding approach utilizing a mixer mill.^49^ Stoichiometric ratios (1 : 2) consisting of 45 mg of myricetin and 35 mg of nicotinamide as previously determined were combined in a 10 ml stainless steel grinding jar with two 7 mm diameter stainless steel grinding balls as milling media. The milling time was set to 10, 20, and 60 min at an operating oscillation frequency of the mill of 1800 rpm (30 Hz). The resulting solids were dried in a fume hood for 24 h at ambient temperature.^49^

##### 2.1.1.2 Bottom-up techniques

Bottom-up approaches grow NCCs from solution *via* two crucial steps *viz.*, nucleation and crystal growth. Nucleation is particularly important to produce uniform NCCs. The larger the increase in nucleation rate the more the number of nuclei formed from the supersaturated solution. This then leads to a reduction in supersaturation resulting in less growth for each nucleus as a result.^50^ If a great number of nuclei are generated concurrently during nucleation, a narrow polydispersity index is expected.^50^

Therefore, it is essential to promote rapid and homogeneous nucleation when using a bottom-up process. Mixing of the drug solution and an anti-solvent is generally achieved with conventional mixing equipment, *e.g.* magnetic stirring or use of an agitator blade.^51^ Nucleation can be prompted by mixing with an anti-solvent or removal of solvent^36^ or introduction of sonic waves to induce sonoprecipitation.^51,52^ Sonoprecipitation been the most commonly used technique to produce NCC.^49,53–55^

For instance, Sander *et al.*,^53^ successfully utilised sonoprecipitation manufacture caffeine NCCs. The authors report using a single-solvent approach in which 60 mg caffeine and 48 mg of 2,4-dihydroxybenzoic acid (DHBA) were added in 7 ml and 242 μl acetone, respectively.^53^ The solutions were rapidly injected into 200 ml of hexane at approximately 0 °C and sonicated for 15 s in a cleaning bath. A two-solvent approach using the same procedure was also performed in the single solvent approach 125 mg of caffeine and 99 mg DHBA were dissolved separately in 1 ml chloroform and 600 ml acetone and rapidly injected into 100 ml of hexane at approximately 0 °C. In these experiments Span® 85 at a concentration of 5% w/v in hexane was utilised as stabiliser.^53^

Similarly, Huang and co-workers^54^ manufactured a phenazopyridine-phthalimide NCC suspension. This was achieved by dissolving separately dissolving phenazopyridine and phthalimide in 2 ml of dimethyl sulfoxide (DMSO). Subsequently, the individual solutions were rapidly injected into 50 ml 0.4% w/v SDS aqueous solution at approximately 2 °C using ultrasonic conditions. After 15–30 s, the authors reported the formation of NCC.^54^

Liu *et al.*,^49^ also formulated NCC utilising a bottom-up approach. Bottom-up NCC were produced *via* a one-solvent solution approach in which 311 mg myricetin and 402 mg nicotinamide were dissolved separately in 7 ml and 3 ml methanol, respectively.^49^ This was followed by rapid injection of the into a conical flask at 0 °C. Ultrasonic energy was applied to the mixture 30 min using an ultrasonic processor set at a frequency of 40 kHz with a power output of 50 W. Thereafter, the low-temperature control was withdrawn but ultrasonication was maintained. The resulting precipitate was removed after 10, 20, and 30 min, filtered and dried for 24 h at room temperature.^49^ The NCCs produced using the bottom-up approach yield smaller NCCs compared to the top-down method and further exhibited better enhanced aqueous solubility when compared to micronized co-crystals and top-down NCCs.^49^

Witika *et al.*, manufactured lamivudine-zidovudine NCCs *via* pseudo one-solvent sonochemical precipitation in which both co-crystal forming components were dissolved separately in different solvents so that each solvent served as an anti-solvent for the other, *in situ*.^55^ In these experiments, AZT and 3TC were dissolved in methanol and water, respectively after saturation studies had been performed. The separate solutions were rapidly injected into a pre-cooled conical flask incubated in an ice bath and sonicated for 20 min.^55^

#### 2.1.2 One-pot synthesis

The synthesis of many nanomaterials involves multi step processes to obtain products with desirable CQAs. Some steps involve secondary extrusion or sonication to reduce particle sizes as with vesicular drug delivery systems. NCCs combine the ability to produce nanomaterials with desirable CQAs in one-step processes.

The use of simple top-down techniques such as wet media milling,^42–44,48^ neat grinding^43,49^ and HPH^41^ are capable of producing NCCs within the nanoscale with narrow particle size distributions. Similarly, a one-pot synthesis technique for the development of bottom-up NCCs have been reported.^49,53–55^

These studies demonstrate the ease with which NCCs can be developed and have scale up potential. Furthermore, utilising a one-pot synthesis method could potentially result in a relatively inexpensive production process translating into more affordable medicines.

### 2.2 Critical material attributes (CMAs)

Critical material attribute (CMAs) are defined as a material with variability having an impact on the CQAs and therefore should be monitored or controlled to ensure that the desired QTPPs are attained.^30^

Often, NCCs, much like nanocrystals, are easy to synthesise, but their stability and the selection of stabiliser(s) is the most challenging and critical step.^56^ NCCs are stabilised by the use of either polymers or surfactants. The polymers and/or surfactants used impart steric and/or electric stabilisation there by preventing agglomeration of molecules arresting the development growth at the nanoscale.^56^

In many instances the choice of stabiliser can have a profound effect on the CQAs of NCCs. For instance, it was demonstrated that the choice of stabiliser could have a significant effect on CQAs of NCCs when using a bottom-up technique. The aim was to demonstrate the role of different surfactants such as Tween® 80, Span® 80, SDS and α-tocopheryl polyethylene glycol succinate 1000 (TPGS 1000) and their effect on the CQAs *viz.*, particle size (PS), the polydispersity index (PDI) and zeta potential (ZP) for the reported NCCs.^55^ While all the investigated stabilisers produced NCCs, the concentration of stabiliser influenced the CQAs. Similarly, the nature of the stabiliser (ionic or non-ionic) had a profound effect particularly on the ZP of the NCCs.^55^

Huang *et al.*, also investigate the possibility of manufacturing NCCs using different stabilisers. The NCCs were prepared using, PVP, hydroxypropylmethyl cellulose (HPMC), hydroxypropyl cellulose (HPC), TPGS 1000, Tween® 80, SLS, poloxamer 188 and poloxamer 407. Of these PVP and HPMC were not able to form nanosuspensions and from the remaining stabilizers HPC, Tween® 80 and poloxamer 407 were selected for further long-term stability at different storage temperatures.^45^ The results demonstrated the flexibility of the stabilisers that can yield ideal NCC based on the desired QTPP.^45^

These studies demonstrate how the different stabilisers can be utilized to get similar CQA but could have a different effect on the QTPP with regards to taste, appearance or overall shelf life.

### 2.3 Enhancement of critical quality attributes (CQA)

Critical quality attributes are described as physical, chemical, biological or microbiological properties or characteristics that should be within an appropriate limit, range, or distribution to ensure the product is of desired quality.^30^

Pharmaceutical nanocrystals and co-crystals present a promising and emerging approach to modulate the performance of pharmaceuticals *viz.*, physical stability, chemical stability, mechanical properties, optical properties, release profiles, bioavailability and therapeutic effect.^57,58^

#### 2.3.1 Solubility enhancement and improvement of dissolution rate

It has been extensively studied and demonstrated that the atomic packing in the unit cell and crystal lattice have a direct impact on the physicochemical properties of crystalline materials.^59,60^ Among the properties affected is the solubility and solubility rate and these form, in part, the basis for co-crystal solubility enhancement. Nanonisation of materials is another technique well known to increase the solubility of crystalline materials.^6,58^ NCC combine these two techniques and could possibly have greater effect on the solubility of materials than either technology.

The solubility improvement of NCC over nanocrystals and co-crystals was demonstrated by Pi *et al.*, for baicalein and nicotinamide utilizing a top-down technique. The results obtained from *in vitro* and *in vivo* assessments showed BE-NCT NCC had the highest dissolution rate compared to BE-NCT co-crystals, BE-nanocrystals, and BE coarse powder.^41^ The dissolution rate of the BE-NCT showed a 2.17-fold increase to the coarse BE powder compared to a 2.01-fold and 1.74-fold increase for the BE-nanocrystals and BE-NCT co-crystals, respectively.^41^ The *in vivo* evaluations to investigate the pharmacokinetic factors post oral administration on BE-NCT NCC, BE-nanocrystals, BE-NCT co-crystals and BE coarse powder showed that there was a 6.02-fold increase of AUC for the BE-NCT NCC which was higher when compared to the 3.32-fold and 2.87-fold increase with the BE nanocrystals and BE-NCT co-crystals, respectively.^41^

Bhandari *et al.*, demonstrated a significant increase in solubility when a bottom-up technique was utilised to manufacture ezetimibe NCC.^61^ The NCC dissolution profile demonstrated 18.8-fold increase in the dissolution efficiency. The results demonstrate feasibility of nano co-crystallisation using a suitable co-former has the ability to significantly increase the dissolution properties of API.

NCC have also been developed to enhance the solubility of sulfamethazine and 4-aminosalicylic acid.^62^ The authors report manufacturing NCC using HPH and ultrasonication techniques. In these experiments, the HPH produced NCC, while the high-power ultrasound crystallisation method only yielded co-crystals with a mean size in the micro-size range. The NCC produced by HPH significantly improved the dissolution rate compared to micro-size cocrystals and even more compared to pure API. Furthermore, the NCC demonstrated stability over 6 months storage periods.^62^

Similar solubility enhancements of the NCC over nanocrystal and co-crystal formulations have been demonstrated for furosemide when nano co-crystallised with caffeine and carbamazepine (CBZ) nano co-crystallised with saccharin (SAC).^42^ The CBZ–SAC NCC suspension exhibited rapid dissolution and a dissolved concentration that was ∼1.5 times higher than that of the CBZ nanocrystal. The furosemide nano co-crystal demonstrated similar superiority in dissolution rate and overall dissolution profile when compared the micro co-crystal and unmilled suspensions.^42^

These data demonstrate how NCC have a promising ability to improve the solubility and dissolution rate of biopharmaceutics classification system (BCS) class II and IV molecules.

While NCC could potentially be used to improve many other CQA associated with co-crystals^63^ and nanocrystals,^64^ there are no specific studies highlighting such improvements and as such, would present a vast amount of opportunities for NCC development.

**Fig. 2:**
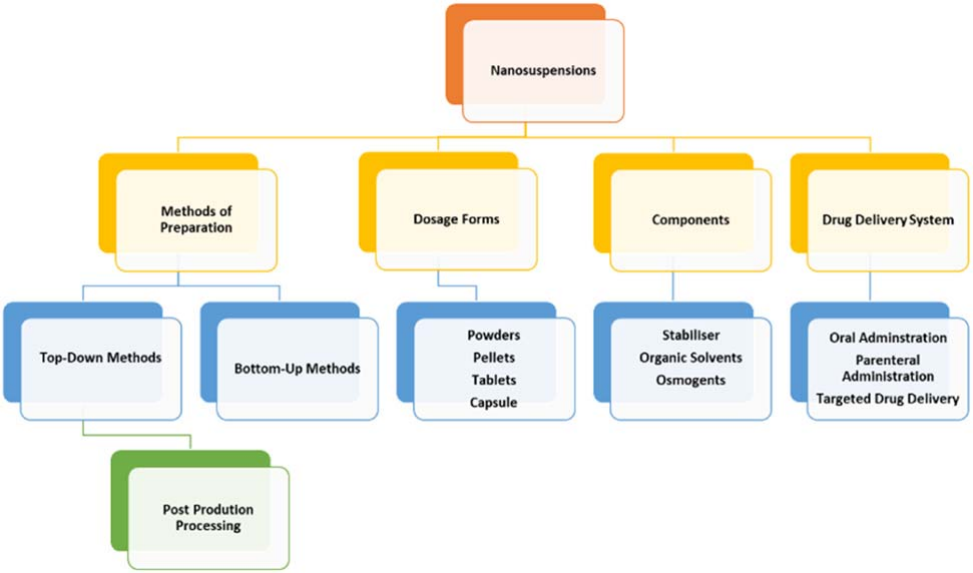
Summary of Strengths of NCC. Reproduced with modification from ref. 1 with permission from Springer Nature and in accordance to Creative Commons Agreement (https://creativecommons.org/licenses/by/4.0/).

A summary of the strengths by component of NCC is provided in Fig. 2.

## 3 Weaknesses

### 3.1 Critical material attributes

Despite the many properties that make NCC such a desirable technology, there remain some aspects that remain cardinal and may delay their potential for successful development and translation into marketable products.

#### 3.1.1 Co-former selection

Due to the hybrid nature between co-crystals and nanocrystals, NCC still have the potential to be unsuccessful if a suitable co-former is not identified. The process of co-former selection is identical to that of co-crystal manufacture and largely determines how effective the technology will be.

The primary approach considered tactless and involves the screening of the co-formers from a library of substances which are GRAS.^65^ A different and possibly more useable approach is to utilise a supramolecular synthon approach which makes use of Cambridge Structural Database (CSD) for statistical analysis of data. This approach requires a detailed appreciation of supramolecular chemistry and the functional groups present in API of choice.^65^ The use of the Hansen Solubility Parameter (HSP) approach is also used to predict the miscibility and co-crystal formation by using group contribution method and to calculate partial solubility parameters and Van Krevelen–Hoftyzer, Bagley and Greenhalgh approaches to predict miscibility.^65,66^ Often this approach is only capable of in predicting miscibility. However, all co-formers which are predicted may not be miscible making this only a theoretical basis mostly useful for short listing potential co-formers prior to exhaustive laboratory screening experiments. This can lead to greater efficiency in co-crystal screening programs.^65^

Generally, the use of knowledge about hydrogen bonds, synthon chemistry and HSP may assist in design and analysis of co-crystals. However, the prediction of whether co-crystallization will occur is not possible and must, at present, be answered empirically. This directly has implications on the possibility of developing NCC as the primary requisite to forming NCC is API being able to co-crystalise.

#### 3.1.2 Stabiliser selection

Much like in the case of co-former selection, the selection of a suitable stabiliser remains one of the weaknesses of this technology.^67–69^

Huang *et al.*, demonstrated that in some cases readily available polymers may not yield the NCC as expected. The study demonstrated that polyvinylpyrrolidone (PVP) and hydroxypropyl methyl cellulose (HPMC) were not able to form nanosuspensions and only hydroxypropyl cellulose (HPC), Tween® 80 and poloxamer 407 were capable of imparting stability to the NCC suspensions.^45^ The results demonstrated the flexibility of parting stability to the NCC suspensions. The study did not provide a specific reason for the failure of the stabilisers to stabilise the technology.^45^

This demonstrates that the selection of stabiliser is based primarily on searching a library of commonly used stabilisers and using a trial-and-error approach to achieve NCC with desirable CQA.

### 3.2 Quality target product profile of the NCC final product

It remains highly desirable to produce NCCs with the potential to meet end user requirements. These include but are not limited to long term stability, high efficacy, convenient dosing schedule and easy administration. NCC may be affected by many of the potential weakness of either co-crystals or nanocrystals.

#### 3.2.1 Stability

The stability of final product is a major quality looked for in a formulation. With regards to NCC, there are many potential sources of instability that could exist in arise before the final formulation gets to the end user. It is, therefore, very important to take these potential concerns when considering formulating NCC.

##### 3.2.1.1 Final dosage form stability

The unique characteristics of drug nanocrystals have enabled their extensive application in various dosage forms including oral, parenteral, ocular, pulmonary, dermal and other specialized delivery systems.^33,38,70–72^ As successors to nanocrystals, NCC have the same advantages and could possibly be applied as in similar circumstances.

#### 3.2.2 Agglomeration of NCCs

Due to their small size, it is expected that the large surface area of NCC would create high total surface energy, which is thermodynamically unfavourable.^73^ Thus, the NCC will tend to agglomerate to minimise the surface energy. Agglomeration can result in a variety of issues for NCC including rapid settling/creaming, crystal growth and inconsistent dosing.^73^ Consequently, the use of a suitable stabiliser is required to wet the NCC surfaces and prevent agglomeration.^74^

Even though different dosage forms may share some commonality regarding stability concerns such as sedimentation, particle agglomeration or crystal growth, their effects on drug products are not standard.

For example, particle agglomeration could be a major concern in pulmonary drug delivery as it affects deposition quantities and deposition site along the respiratory tract and consequently formulation effectiveness. Conversely, agglomeration in formulations intended for intravenous administration can cause blood capillary blockage and obstruct blood flow.^73^ Additionally, the selection of stabilisers is also closely related to dispersion medium, dosage form and strictly governed by FDA regulations.^73^ Presently, there is a huge number of stabilisers approved for oral drug delivery but only a limited number are approved for inhalation.

#### 3.2.3 Sedimentation

In suspension, NCC can either settle down or cream up in the formulation medium depending on their density relative to the dispersion medium. According to Stoke's law, the sedimentation rate of particles in suspension is closely related to particle size, medium viscosity and density difference between medium and dispersed phase.^75^ In the manufacture of NCC, a reduction of particle size forms a critical aspect in manufacture and forms the most commonly utilised strategy to reduce particle settling. Similarly, the use of use of thickening agents to increase the viscosity of the dispersing media as well as matching NCC drug particle density to that of the dispersion media have also been utilised.^75,76^ While all these strategies are utilised, there is a possibility of nanosuspensions forming a deflocculated suspension, in which particles settle independently as minute size units resulting in a slow sedimentation rate. However, tightly packed sediment, known as caking,^77^ is typically formed due to the pressure applied on each individual particle. The redispersion of this cake is almost impossible even on agitation^75,77^ and possess a stability concern to the final drug products performance. Alternatively, the nanosuspensions could form flocculated suspensions in which the clustered particles settle as loose aggregates instead of as individual particles.^75^ The loose aggregates have a larger size compared to the single particle, and thus higher sedimentation rate. The loose structure of the rapidly settling flocs contains a significant amount of entrapped medium and this structure is preserved in the sediment. In contrast to, the deflocculated suspension, the flocs formed are easily redispersible and have a lesser effect on the final performance of drug product performance.

#### 3.2.4 Crystal growth

Particle size and polydispersity indices in colloidal suspensions increase as a result of Ostwald ripening, commonly known as crystal growth. Solubility dependency on particle size is the key idea of Oswald ripening. According to the Ostwald–Freundlich equation,^8^ smaller particles have a higher saturation solubility than bigger ones, which results in a gradient in drug concentration between small and large particles. As a result, molecules move from areas of smaller particles with higher drug concentration to areas of bigger particles with lower drug concentration. As a result, a supersaturated solution forms around the big particles, causing the medication to crystallize on top of them. The drug molecules dissolve from the small particles into the bulk medium due to the diffusion process, which produces an unsaturated solution around them. The drug molecules dissolve from the small particles into the bulk medium because of the diffusion process, which produces an unsaturated solution around them. The small particles are dissolved and then deposited onto the larger particles as the diffusion process continues. The Ostwald ripening can be prevented by reducing the gradients in drug concentration and saturation solubility within the medium, which is possible with a lower polydispersity index.^1^ This may help to explain why for nanosuspensions with homogeneous particle size, Ostwald ripening is not a significant concern.^6,9^ As long as they don't increase the solubility of the API, stabilisers may help reduce Ostwald ripening.^10,11^ The stabilisers, which are adsorbed on the NCC's surface, can lessen the interfacial tension between the solid particles and liquid medium, inhibiting Ostwald ripening as a result. Ostwald ripening is further influenced by solubility, temperature, and mechanical agitation.^11^

#### 3.2.5 Stability on solidification

In many cases the choice of the NCC in the final product is in solid state as many stability concerns are eliminated in the solid. The most utilised process to obtain solid dry powders are freeze drying and spray drying.^38,71,78,79^ These dry powders are intended for reconstitution into nanosuspensions prior to administration and as such there is a requirement to prevent the of growth or agglomeration of NCC during the drying. In this way, the features associated with nanosizing such as rapid dissolution following the reconstitution are maintained and avoidance of arterial or venous blockage in the event of IV administration. To prevent this instability in the solidification process, the addition matrix formers, such as mannitol, sucrose and cellulose prior to drying is commonly utilised.^78^

## 4 Opportunities

The use of NCCs present a variety of opportunities. The technology is not vastly explored allowing for the utilization of many gaps in present options. As this technology is a combination of co-crystals and nanocrystals, it allows for the exploration and commercialization of products based on successes of either technology with potential added benefits.

### 4.1 Combination therapy

Co-crystals have the ability to deliver two or more API that could potentially have synergistic effect.^80^ At the very least, NCC technology allows the delivery of two API in one dosage form. The possibility of delivering multiple payloads is particularly attractive in the management and treatment of diseases that require than one API such as HIV/AIDS, tuberculosis, cancer and malaria among others.

This has been demonstrated in the manufacture of an NCC formulation to potentially delivery both lamivudine and zidovudine for the management of HIV/AIDS.^48,55,81,82^

Similarly, Pi *et al.*, demonstrated the possibility of combining the botanical therapeutic baicalein and nicotinamide to improve the solubility of the baicalein.^41^ The authors were able to manufacture a formulation capable of increasing the bioavailability of the baicalein while also being able to deliver nicotinamide.^41^

Similarly, Mohammad and colleagues^83^ created NCCs of the anticancer drug paclitaxel with the anti-alcohol drug disulfiram to increase the anticancer drug's efficiency by reversing MDR. According to reports, disulfiram inhibits P glycoprotein (MDR protein) and improves the tumour cells' sensitivity to the payload. When compared to pure paclitaxel NPs, the NCCs of paclitaxel and disulfiram showed a considerable (14-fold greater) absorption in taxol-resistant lung cancer (A549/TAX) cells. In addition, NCCs were found to have greater apoptosis (5-folds) and exceptional cytotoxicity (low IC_50_; 7-folds lower) than free paclitaxel.^83^

These experiments demonstrate the potential for NCC to develop into a sustainable multi-drug delivery tool in pharma and nutraceutical applications.

### 4.2 Extension of intellectual property

The development of NCC have the added benefit of being considered new drug entities much like co-crystals.^84,85^ To be granted a patent, a pharmaceutical co-crystal, like the claimed subject matter of any patent application, must be useful, novel, and non-obvious.^86^ A co-crystal is a distinct solid-state material with a distinct and unpredictable structure and physical property profile.^87^ Furthermore, co-crystallisation of API molecules has a reasonable chance of obtaining patent protection in addition to that of the parent API because they are new chemical entities for which the design and preparation involve several non-obvious elements, and they generally exhibit novel and useful properties.^88,89^

NCC can be considered to fit into this description and would allow for the extension or registration of a patent, specifically when a manufacturing method to use to nano size the co-crystal with a specific set of stabilisers is considered.

Moreover, the regulatory aspects of nanocrystals also allow for the patenting of a NCC formulation. The nanocrystal regulatory experience as well as a pathway to approval appears to be established in a conventional framework.^90^ NCC are stabilised by use of stabilisers adsorbed onto the surface of the particle surface. Furthermore, in the future, functionalisation of the NCC surface, *e.g.*, for targeting purposes, may be applied. These modifications could be considered as an “engineered” surface on the parenteral administration of coated nanomedicine products.^91^

### 4.3 Additive manufacturing

The possibility of additive manufacturing allows for the development of a product with desired/improved QTPP. NCC, like co-crystals and nanocrystals, can be further manufactured into desirable oral, parenteral or other dosage forms to suit the needs of the end user.^92,93^

NCC have the potential for additive manufacturing based on the successes of further processing of nanocrystals and co-crystals. It has been demonstrated on occasion that nanocrystals can be processed into a convenient solid oral dosage form and the CPP and CMA attributed to this additive manufacture studied.^94^

Similarly, nanocrystal parenteral formulations have been developed for the delivery of long acting antiretroviral drugs rilpivirine,^95,96^ cabotegravir^96,97^ and maraviroc.^98^ These demonstrate the possibility of additive manufacturing of nanocrystals that can be extrapolated to their newer NCC counterparts.

Nanocrystals have been demonstrated to be capable to be further developed exploiting combinatory techniques to exploit multiple advantages. For instance, nanocrystals have been further developed by the utilisation of 3D printing to further develop the technology and derive increased utility.^99–101^ Furthermore, the prospect of loading nanomaterials in nanofibers presents an additional opportunity for further manufacture.^102^ These could be extrapolated to NCC for potential additive manufacturing.

While additive manufacturing has not been demonstrated much specifically for NCC, it has been attempted to further develop NCC of antiretroviral drugs into a stimuli responsive gel allowing for potential sustained/controlled release of the payload.^82^ It was reported that the stimuli responsive carrier was able to sustain the release of the payload for up to 168 hours *in vitro*. The authors do, however, maintain that *there* is a need for more studies pertaining to the actual implications of these data when applied to *in vivo* models.^82^

### 4.4 Improving safety profile

The use of NCC provides an opportunity to target “hard-to-reach” areas achieving better targeting and safety over conventional APIs synthesized. This is achieved by surface modification of the NCC with the appropriate surfactants/polymers. In the manufacture of nanocrystals it has been demonstrated that the choice of stabiliser profoundly has an effect on the safety profile.^103^ The ability of the final technology to provide targeted drug delivery ranges from the effect of the stabiliser on the particle size^104,105^ to the active targeting capabilities of the stabiliser.^106,107^

### 4.5 Flexibility of quality target product profile (QTPP)

NCC have the potential to be utilized in a variety of applications. Being combination of nanocrystal and co-crystal technology, NCC can be adapted much like either of technologies. NCC have the potential to exhibit the advantages associated with co-crystals regarding the summation of the individual components resulting in possibilities such as faster onset of action, taste-masking and dosage form flexibility as well as the advantages of nanocrystals including varied dosage forms^22,58^ and routes of administration^108^ and faster onset of action.^95,98,109,110^

Furthermore, the use of release modifying polymers as coating materials, modification of particle size distribution, particle stability, storage in the tissues, and in some instances a slow prodrug release have all been in implicated as mechanisms for controlled drug release in nanocrystals.^108^ Utilising these strategies could result in producing NCC with controlled release properties.

**Fig. 3:**
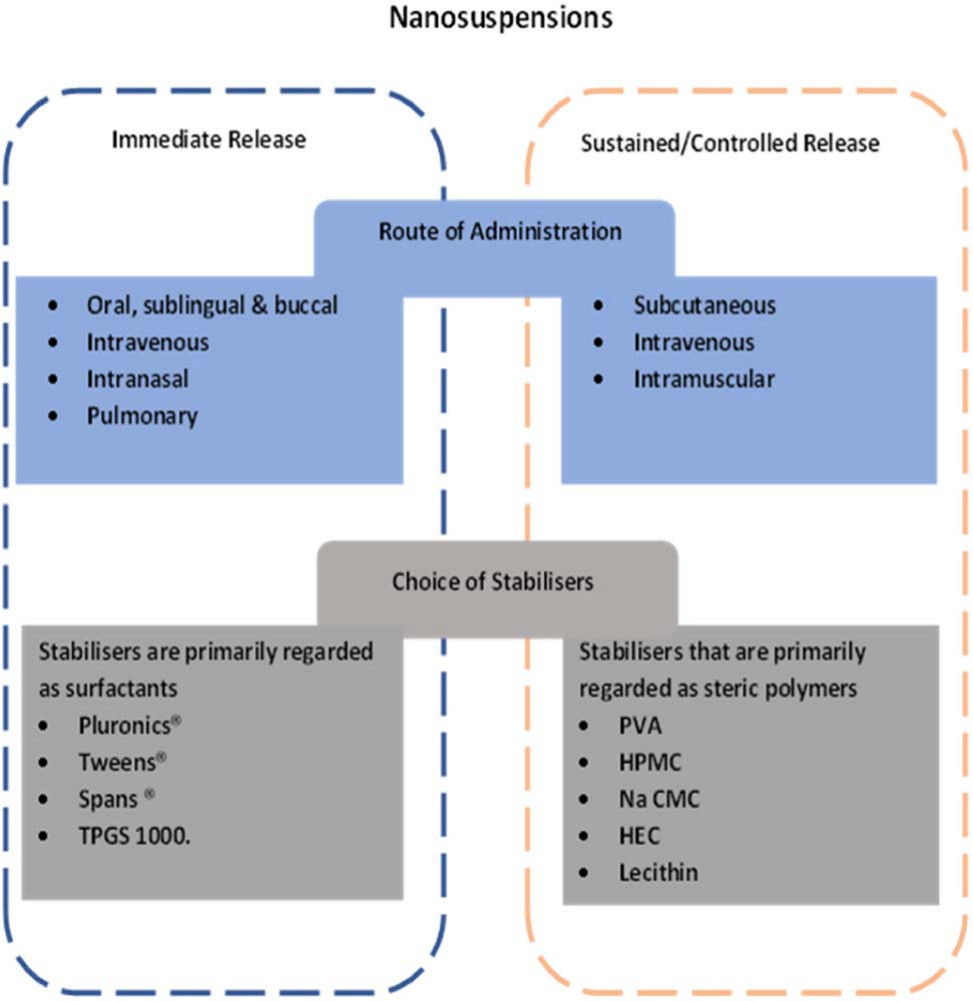
Summary of potential approaches for development of NCC for immediate release or controlled release.

A summary of the potential opportunities related NCC derived from their similarity to nanocrystals is provided in Fig. 3.

### 4.6 Improvement of the pharmacodynamics and pharmacokinetics of potent APIs

One of the primary goals when developing nanomedicines is improve the chances of API reaching the target sites by evading rapid liver/renal clearance. Consequently, the time the API spends in circulation is expected to be extended in the body and, therefore, maximising the chance of eventually reaching the target site. Elimination patterns depend on the nanomaterial itself *viz.*, size, shape, electric charge, stealthiness. This is due to the physicochemical properties of the nanomaterial having a considerable impact on interactions with plasma proteins and thus recognition by the immune system.^111,112^

NCC could potentially exploit these possibilities related to other nanomedicines particularly attributed to their size and shape. Witika *et al.*, demonstrated that different techniques could result in different shapes of NCC.^48,81^ While the effect of shape was not investigated in the studies, it is well known that nanomaterial shape could potentially have a direct effect on efficacy and/or toxicity of the technology.^113,114^ This effect is particularly observed in cancer nanotherapy.^112^ The two main aspects of this expected improvement are related to the passive tumour targeting (EPR effect) and the possibility for active tumour targeting.

The main outcomes of these are the potential for increased efficacy which would result in reduced doses for many anti-cancer drugs. In addition, the possibilities of reduced toxicities is also one that brings excitement.^112^ This has been demonstrated for nanocrystals^115–118^ and can be extrapolated to NCC.

### 4.7 Stability enhancement

Many APIs have inherent stability concerns with regards to hygroscopicity and deliquescence. These can make processing and storage of medicines a big challenge. Co-crystallisation has been used to overcome these challenges.

For instance, ionic co-crystals have been developed to overcome the deliquescence of lithium chloride and lithium bromide. The researchers utilised α-d-glucose as a co-former to overcome this shortcoming.^119^

Similarly, co-crystallisation has been utilised to improve the relative humidity associated stability of theophylline.^120^ The authors demonstrated the use of oxalic acid to be capable of increasing the stability of theophylline by specifically avoiding the formation of a hydrate after co-crystallisation. This was a follow up study after the same authors were able to increase the stability of caffeine using the same co-former with regards to hydrate formation.^121^

There are many other examples of co-crystals being utilised to provide stabilisation against heat, moisture, light, and oxygen.^122^

As NCC are nanosized co-crystals, there is a potential for this technique to be utilised in much the same way.

### 4.8 Technology transfer from academia to industry

Drug nanocrystals comprise unique drug delivery platforms playing a significantly important and distinctive role in drug delivery and as such, many academic researchers are investing plenty time and money in developing nano co-crystal (NCC) products. NCCs are considered as new drug products by the US Food and Drug Administration (FDA) and not as generics since their pharmacokinetic profile is not bioequivalent to other solubilized forms of the same drug administered at the same dosage. Therefore, NCCs can be patented as a new drug entity that can provide industry with product line extensions for existing drug formulations with a more rational formulation design. Hence, technology transfer from NCC research in academia to industry presents a great opportunity to ensure research advancements in this area eventually have a clinically meaningful impact.

Technology transfer of NCCs as drug delivery formulations can be a complex process that involves many stakeholders and several factors that can impact implementation. For example, extensive feasibility studies are needed to assess the CPPs of various NCC prototypes as well as their critical quality attributes CQAs after scale-up are validated within a rationale formulation design. This can include extensive validation requirement (and specifications) to their enhanced solubility and dissolution, improved bioavailability, food effects, safe dosing, safety, efficacy and tolerability as some of the advantages that the drug delivery technology provides.

Furthermore, not all synthesis methods are amenable for transfer and the economic feasibility is critical to the transfer flow. However there still remains much promise as several nanocrystal based products have been licensed with regulatory approval either already with market authorization or are in preclinical stages of development. Examples include products such as Semapimod® (guanylhydrazone), Paxceed® (paclitaxel), Heralux® (thymectacin), Nucryst® (silver), Rapamune® (sirolimus), Emend® (aprepitant) and TriCor® (fenofibrate).

## 5 Threats

### 5.1 Translation into marketable products

For every product that is developed there is an obvious need to translate the use of the product to the clinic. The NCC technology is one that is generally applied to compounds that have a low developability. NCC technology can generally be considered delayed to market for similar reasons as nanocrystals were initially delayed. These concerns initially were centred around companies lacking experience and capabilities to cover the whole scale-up and clinical manufacturing process which is quite sophisticated for nanocrystal preparations^90^ and would ideally be more complex for NCC. This would entail the technology will have to compete with other enabling techniques to formulate drug candidates of low developability, which can more readily be performed in-house.^90^

**Fig. 4:**
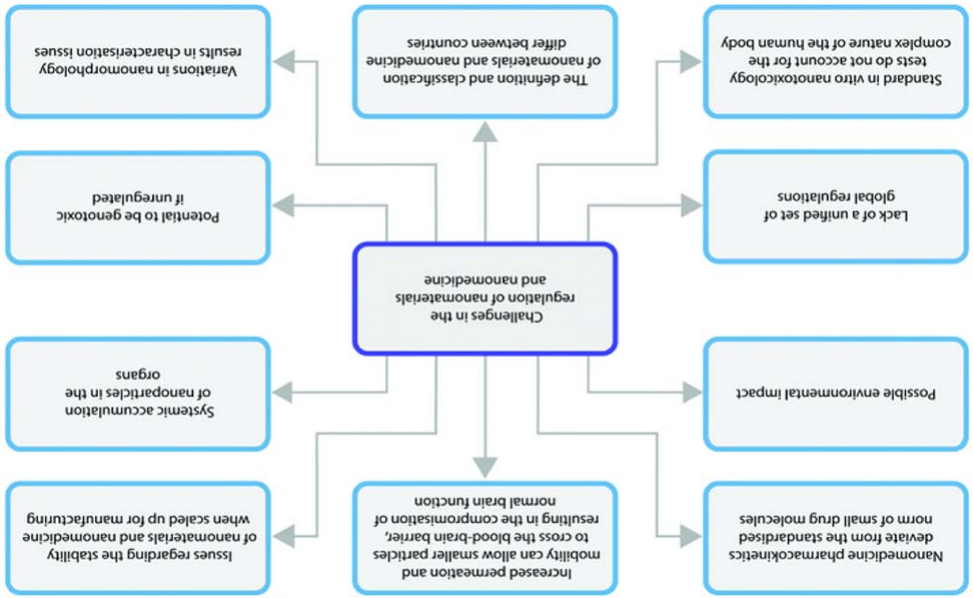
Schematic illustration highlighting the major challenges in nanomaterials regulation. Reproduced without modification from ref. 123 with permission from the Royal Society of Chemistry in accordance with the Creative Commons Agreement (https://creativecommons.org/licenses/by-nc/3.0/).

This notwithstanding, NCC still face the same challenges as all other nanomedicines in the regulatory pipeline.^123^ A summary of the regulatory aspects is provided in Fig. 4.

### 5.2 Contamination

The development of NCC can be by either top-down or bottom-up methods. While these techniques are relatively straightforward, they present with the possibility of contamination from the milling media in top-down methods^48,124,125^ in the top-down method and the possibility of the concentration of residual solvents exceeding acceptable limits in bottom-up techniques.^126^ This could be a threat to the development of the technology as the purity of the NCC formulation could be compromised.

### 5.3 Other established nano drug delivery systems

The development of NCC is also under threat from other more established and translated nano drug delivery systems like liposomes, micelles or solid lipid nanoparticles that have already entered the market and most companies have the capacity to utilise already existing infrastructure and personnel. This was particularly evident in the rapidity with which lipid based nanoparticles were utilised in the recent coronavirus disease 2019 vaccine development^127,128^ and may provide an insight to future nanomedicines and vaccines development.^127^ Furthermore, many of the advantages associated with NCCs such as enhanced solubility and improved stability can also be achieved by the use of the aforementioned established drug delivery systems.

## 6 Future perspectives

The prospects of nanosuspensions in the form of NCC are promising as they can contribute as an alternative tool for drug development scientists to circumvent many formulation and drug delivery challenges specifically with troublesome API. The field on NCC is still in its infancy and can only grow based on the successes of the several published.

One really exciting area for this technology, which can be extrapolated from nanocrystal technology, is in the advancement in biotechnology and aid of modification tools such as antibody–drug conjugate and nanobodies with the potential to create simultaneous administration of API and high concentration monoclonal (mAb) and biosimilar products. These would be expected to have properties enhanced biopharmaceutical and safety characteristics. It was recently demonstrated that a biologically active, nanocluster dispersion of antibodies in solution using carbohydrate stabilisers can be manufactured with the potential enable patient self-administration by subcutaneous injection of antibody therapeutics.^129^ Future development of enabling technologies like NCC can provide technical solutions to many formulation challenges currently faced by protein and peptide-based API.

## 7 Conclusions

Nanocrystallisation and co-crystallisation are two forms of crystal engineering generally used for improvement of physicochemical properties of many APIs generally in the BCS class II and IV. They can be considered as one of the top options for formulation of intractable hydrophobic payloads constrained by high molecular weights, highly positive log *P* values, high melting points and high dose. Orthodox size reduction techniques such as wet media milling and HPH for top-down and cold sonoprecipitation, emulsion-solvent evaporation, solvent diffusion and microemulsion techniques for bottom-up methods can be successfully used to combine nanocrystallisation with co-crystallisation and benefit from a synergy between the techniques. The methods of manufacture are easily scalable to produce nanosuspensions with more than one payload. The NCC technology can be utilised to improve the bioavailability of API as a consequence of increased saturation and intrinsic solubility. Moreover, NCC can be used to impart appreciable mucoadhesivity by adaptability for surface modification on the co-crystal surface. The surface of the co-crystal can further be modified for on-target drug delivery, reducing adverse effects.

It is also cardinal to note the flexibility of NCC with regards to routes of administration and potential to achieve drug delivery in ways nanosuspensions or co-crystals were incapable. It does remain, however, to be seen how best this novel technology can be explored to overcome the shortcomings of its predecessors.

## Author contributions

Dr Bwalya A. Witika (conceptualisation, drafting and paper writing); Prof Yahya E. Choonara (drafting and reviewing the paper); Prof Patrick H. Demana (drafting and reviewing the paper).

## Conflicts of interest

There are no conflicts to declare.

## Supplementary Material

## References

[cit1] Jacob S., Nair A. B., Shah J. (2020). Biomater. Res..

[cit2] Blagden N., de Matas M., Gavan P. T., York P. (2007). Adv. Drug Deliv. Rev..

[cit3] Shah D. A., Murdande S. B., Dave R. H. (2016). J. Pharm. Sci..

[cit4] Bajaj A., Rao M. R. P., Pardeshi A., Sali D. (2012). AAPS PharmSciTech.

[cit5] Batisai E. (2021). ChemistryOpen.

[cit6] Couillaud B. M., Espeau P., Mignet N., Corvis Y. (2019). ChemMedChem.

[cit7] Subramanian S., Zaworotko M. J. (1995). Can. J. Chem..

[cit8] Desiraju G. R. (1991). J. Appl. Crystallogr..

[cit9] Desiraju G. R. (1995). Angew. Chem., Int. Ed..

[cit10] Bolla G., Sanphui P., Nangia A. (2013). Cryst. Growth Des..

[cit11] Gagniere E. , Mangin D., Veesler S. and Puel F., Co-crystallization in Solution and Scale-up Issues, Royal Society of Chemistry, London, 2012

[cit12] Aakeröy C. B., Fasulo M. E., Desper J. (2007). Mol. Pharm..

[cit13] Aakeröy C. B., Salmon D. J. (2005). CrystEngComm.

[cit14] Aakeroy C. B. , Aakeroy A. and Sinha A. S., Co-crystals: Introduction and Scope, Royal Society of Chemistry, London, 2018, vol. 11

[cit15] Bolton O., Matzger A. J. (2011). Angew. Chem., Int. Ed..

[cit16] Brittain H. G. (2013). J. Pharm. Sci..

[cit17] Brittain H. G. (2011). Cryst. Growth Des..

[cit18] Sekhon B. (2009). ARS Pharm..

[cit19] Gao Y., Zu H., Zhang J. (2011). J. Pharm. Pharmacol..

[cit20] Bethune S. J., Schultheiss N., Henck J. O. (2011). Cryst. Growth Des..

[cit21] Jayasankar A., Reddy L. S., Bethune S. J., Rodríguez-Hornedo N. (2009). Cryst. Growth Des..

[cit22] Müller R. H., Gohla S., Keck C. M. (2011). Eur. J. Pharm. Biopharm..

[cit23] Noyes A. A., Whitney W. R. (1897). J. Am. Chem. Soc..

[cit24] Kipp J. , Wong J., Doty M., Rebbeck C., Microprecipitation method for preparing submicron suspensions, US Pat., 6869617, 2006, p. 2

[cit25] Wang G. D., Mallet F. P., Ricard F., Heng J. Y. Y. (2012). Curr. Opin. Chem. Eng..

[cit26] Salazar J., Ghanem A., Müller R. H., Möschwitzer J. P. (2012). Eur. J. Pharm. Biopharm..

[cit27] Möschwitzer J., Müller R. H. (2006). J. Nanosci. Nanotechnol..

[cit28] Fontana F., Figueiredo P., Zhang P., Hirvonen J. T., Liu D., Santos H. A. (2018). Adv. Drug Deliv. Rev..

[cit29] Junyaprasert V. B., Morakul B. (2015). Asian J. Pharm. Sci..

[cit30] ICH , Pharmaceutical Development Q8 (R2), 2009, vol. 8

[cit31] Ghosh I., Bose S., Vippagunta R., Harmon F. (2011). Int. J. Pharm..

[cit32] Chogale M. M., Ghodake V. N., Patravale V. B. (2016). Pharmaceutics.

[cit33] Merisko-Liversidge E., Liversidge G. G. (2011). Adv. Drug Deliv. Rev..

[cit34] Merisko-Liversidge E., Liversidge G. G., Cooper E. R. (2003). Eur. J. Pharm. Sci..

[cit35] Sinha B., Muller R. H., Moschwitzer J. P. (2013). Int. J. Pharm..

[cit36] De Waard H., Frijlink H. W., Hinrichs W. L. J. (2011). Pharm. Res..

[cit37] Shegokar R., Müller R. H. (2010). Int. J. Pharm..

[cit38] Rabinow B. E. (2004). Nat. Rev. Drug Discovery.

[cit39] Muller R. H., Jacobs C., Kayser O. (2001). Adv. Drug Deliv. Rev..

[cit40] Pi J., Liu Z., Wang H., Gu X., Wang S., Zhang B., Luan H., Zhu Z. (2016). Curr. Drug Deliv..

[cit41] Pi J., Wang S., Li W., Kebebe D., Zhang Y., Zhang B., Qi D., Guo P., Li N., Liu Z. (2018). Asian J. Pharm. Sci..

[cit42] Karashima M., Kimoto K., Yamamoto K., Kojima T., Ikeda Y. (2016). Eur. J. Pharm. Biopharm..

[cit43] Nugrahani I., Auli W. N. (2020). Heliyon.

[cit44] De Smet L., Saerens L., De Beer T., Carleer R., Adriaensens P., Van Bocxlaer J., Vervaet C., Remon J. P. (2014). Eur. J. Pharm. Biopharm..

[cit45] Huang Z., Staufenbiel S., Bodmeier R. (2022). Pharm. Res..

[cit46] Huang Z., Staufenbiel S., Bodmeier R. (2022). Int. J. Pharm..

[cit47] Huang Z., Staufenbiel S., Bodmeier R. (2022). Eur. J. Pharm. Biopharm..

[cit48] Witika B. A., Smith V. J., Walker R. B. (2021). Crystals.

[cit49] Liu M., Hong C., Li G., Ma P., Xie Y. (2016). Nanotechnology.

[cit50] Lu Y., Li Y., Wu W. (2016). Acta Pharm. Sin..

[cit51] Xia D., Gan Y., Cui F. (2014). Curr. Pharm. Des..

[cit52] Dalvi S. V., Yadav M. D. (2015). Ultrason. Sonochem..

[cit53] Sander J. R. G., Bučar D. K., Henry R. F., Zhang G. G. Z., MacGillivray L. R. (2010). Angew. Chem., Int. Ed..

[cit54] Huang Y., Li J.-M., Lai Z.-H., Wu J., Lu T.-B., Chen J.-M. (2017). Eur. J. Pharm. Sci..

[cit55] Witika B. A., Smith V. J., Walker R. B. (2020). Pharmaceutics.

[cit56] Peltonen L., Hirvonen J. (2010). J. Pharm. Pharmacol..

[cit57] Guo M., Sun X., Chen J., Cai T. (2021). Acta Pharm. Sin..

[cit58] Muller R. H. and Junghanns J. A. H., in Nanoparticulates as Drug Carriers, Imperial College Press, London, 2006, pp. 307–328

[cit59] Schultheiss N., Newman A. (2009). Cryst. Growth Des..

[cit60] Datta S., Grant D. J. W., Hall W. (2004). Nat. Rev. Drug Discovery.

[cit61] Bhandari J., Kanswami N., Lakshmi P. K. (2020). J. Young Pharm..

[cit62] Salem A., Tak A., Nagy S., Hagym A., Fruzsina G. (2021). Pharmaceutics.

[cit63] Karimi-Jafari M., Padrela L., Walker G. M., Croker D. M. (2018). Cryst. Growth Des..

[cit64] Chen M. L., John M., Lee S. L., Tyner K. M. (2017). AAPS J..

[cit65] Fukte S. R., Wagh M. P., Rawat S. (2014). Int. J. Pharm. Pharmaceut. Sci..

[cit66] Mohammad M. A., Alhalaweh A., Velaga S. P. (2011). Int. J. Pharm..

[cit67] Van Eerdenburgh B., Vermant J., Martens J. A., Froyen L., Van Humbeeck J., Augustijns P., Van Den Mooter G. (2009). J. Pharm. Sci..

[cit68] Choi J. Y., Yoo J. Y., Kwak H. S., Nam B. U., Lee J. (2005). Curr. Appl. Phys..

[cit69] Li M., Azad M., Davé R., Bilgili E. (2016). Pharmaceutics.

[cit70] Sharma O. P., Patel V., Mehta T. (2016). Drug Delivery Transl. Res..

[cit71] Patravale V. B., Date A. A., Kulkarni R. M. (2004). J. Pharm. Pharmacol..

[cit72] Atia N. M., Hazzah H. A., Gaafar P. M. E., Abdallah O. Y. (2019). J. Pharm. Sci..

[cit73] Wu L., Zhang J., Watanabe W. (2011). Adv. Drug Deliv. Rev..

[cit74] Gao L., Zhang D., Chen M. (2008). J. Nanoparticle Res..

[cit75] Nutan M. T. H. and Reddy I. K., in Pharmaceutical Suspensions: From Formulation Development to Manufacturing, ed. A. K. Kulshreshtha, O. N. Singh and G. M. Wall, Springer New York LLC, 2010, pp. 1–327

[cit76] Ali Y., Lehmussaari K. (2006). Adv. Drug Deliv. Rev..

[cit77] Florence A. T. and Attwood D., in Physicochemical Principles of Pharmacy, ed. A. T. Florence and D. Attwood, Macmillan Education UK, London, 1998, pp. 252–307

[cit78] Van Eerdenbrugh B., Van den Mooter G., Augustijns P. (2008). Int. J. Pharm..

[cit79] Kesisoglou F., Panmai S., Wu Y. (2007). Adv. Drug Deliv. Rev..

[cit80] Thipparaboina R., Kumar D., Chavan R. B., Shastri N. R. (2016). Drug Discov. Today.

[cit81] Witika B. A., Smith V. J., Walker R. B. (2020). Pharmaceutics.

[cit82] Witika B. A., Stander J. C., Smith V. J., Walker R. B. (2021). Pharmaceutics.

[cit83] Mohammad I. S., He W., Yin L. (2018). Pharm. Res..

[cit84] Wang X., Du S., Zhang R., Jia X., Yang T., Zhang X. (2021). Asian J. Pharm. Sci..

[cit85] Garg U., Azim Y. (2021). RSC Med. Chem..

[cit86] Almarsson Ö., Zaworotko M. J. (2004). Chem. Commun..

[cit87] Trask A. V. (2007). Mol. Pharm..

[cit88] Desiraju G. R. (2010). J. Chem. Sci..

[cit89] Yadav A., Shete A., Dabke A., Kulkarni P., Sakhare S. (2009). Indian J. Pharm. Sci..

[cit90] Borchard G. , Drug nanocrystals, in AAPS Advances in the Pharmaceutical Sciences Series, ed. D. J. A. Crommelin, J. S. B. de Vlieger and S. Mühlebach, Springer International Publishing Switzerland, Zurich, 2015, vol. 20, pp. 171–189

[cit91] European Medicine Agency , Reflection paper on surface coatings: general issues for consideration regarding parenteral administration of coated nanomedicine products, 2013, vol. 44

[cit92] Junghanns J. U. A. H., Müller R. H. (2008). Int. J. Nanomed..

[cit93] Müller R. H. , Shegokar R., Gohla S. and Keck C. M., in Intracellular Delivery. Fundamental Biomedical Technologies, ed. A. Prokop, Springer Netherlands, Dordrecht, 5th edn, 2011, pp. 411–432

[cit94] Tuomela A., Laaksonen T., Laru J., Antikainen O., Kiesvaara J., Ilkka J., Oksala O., Rönkkö S., Järvinen K., Hirvonen J., Peltonen L. (2015). Int. J. Pharm..

[cit95] Baert L., van't Klooster G., Dries W., François M., Wouters A., Basstanie E., Iterbeke K., Stappers F., Stevens P., Schueller L., Van Remoortere P., Kraus G., Wigerinck P., Rosier J. (2009). Eur. J. Pharm. Biopharm..

[cit96] Margolis D. A., Gonzalez-Garcia J., Stellbrink H. J., Eron J. J., Yazdanpanah Y., Podzamczer D., Lutz T., Angel J. B., Richmond G. J., Clotet B., Gutierrez F., Sloan L., Clair M. S., Murray M., Ford S. L., Mrus J., Patel P., Crauwels H., Griffith S. K., Sutton K. C., Dorey D., Smith K. Y., Williams P. E., Spreen W. R. (2017). Lancet.

[cit97] Trezza C., Ford S. L., Spreen W., Pan R., Piscitelli S. (2015). Curr. Opin. HIV AIDS.

[cit98] Tatham L. M., Savage A. C., Dwyer A., Siccardi M., Scott T., Vourvahis M., Clark A., Rannard S. P., Owen A., Tatham L. M., Savage A. C., Dwyer A., Siccardi M., Scott T., Vourvahis M., Clark A., Rannard S. P., Owen A., Long M. (2018). Eur. J. Pharm. Biopharm..

[cit99] Germini G., Peltonen L. (2021). Molecules.

[cit100] N'Gatta K. M., Belaid H., El Hayek J., Assanvo E. F., Kajdan M., Masquelez N., Boa D., Cavaillès V., Bechelany M., Salameh C. (2022). Sci. Rep..

[cit101] Pan J. A., Talapin D. V. (2022). Science.

[cit102] Shetty K., Bhandari A., Yadav K. S. (2022). J. Control. Release.

[cit103] Fuhrmann K., Gauthier M. A., Leroux J. C. (2014). Mol. Pharm..

[cit104] Ganta S., Paxton J. W., Baguley B. C., Garg S. (2009). Int. J. Pharm..

[cit105] Gao L., Zhang D., Chen M., Duan C., Dai W., Jia L., Zhao W. (2008). Int. J. Pharm..

[cit106] Müller R. H., Jacobs C. (2002). Int. J. Pharm..

[cit107] Shubar H. M., Lachenmaier S., Heimesaat M. M., Lohman U., Mauludin R., Mueller R. H., Fitzner R., Borner K., Liesenfeld O. (2011). J. Drug Target..

[cit108] Kalhapure R. S., Palekar S., Patel K., Monpara J. (2022). Expet Opin. Drug Deliv..

[cit109] Roy U., McMillan J., Alnouti Y., Gautum N., Smith N., Balkundi S., Dash P., Gorantla S., Martinez-Skinner A., Meza J., Kanmogne G., Swindells S., Cohen S. M., Lee Mosley R., Poluektova L., Gendelman H. E. (2012). J. Infect. Dis..

[cit110] Van't Klooster G., Hoeben E., Borghys H., Looszova A., Bouche M.-P. P., Van Velsen F., Baert L. (2010). Antimicrob. Agents Chemother..

[cit111] Fanciullino R., Ciccolini J., Milano G. (2013). Crit. Rev. Oncol. Hematol..

[cit112] Rodallec A., Fanciullino R., Lacarelle B., Ciccolini J. (2018). Expet Rev. Clin. Pharmacol..

[cit113] Sukhanova A., Bozrova S., Sokolov P., Berestovoy M., Karaulov A., Nabiev I. (2018). Nanoscale Res. Lett..

[cit114] De Matteis V., Rinaldi R. (2018). Adv. Exp. Med. Biol..

[cit115] Hollis C. P., Weiss H. L., Leggas M., Evers B. M., Gemeinhart R. A., Li T. (2013). J. Control. Release.

[cit116] Hollis C. P., Weiss H. L., Evers B. M., Gemeinhart R. A., Li T. (2014). Pharm. Res..

[cit117] Rayahin J. E., Buhrman J. S., Gemeinhart R. A. (2012). Expert Opin. Ther. Pat..

[cit118] Im G. H., Kim S. M., Lee D. G., Lee W. J., Lee J. H., Lee I. S. (2013). Biomaterials.

[cit119] Duggirala N. K., Smith A. J., Wojtas Ł., Shytle R. D., Zaworotko M. J. (2014). Cryst. Growth Des..

[cit120] Trask A. V., Motherwell W. D. S., Jones W. (2006). Int. J. Pharm..

[cit121] Trask A. V., Jones W. (2005). Top. Curr. Chem..

[cit122] Liu L., Wang J. R., Mei X. (2022). CrystEngComm.

[cit123] Foulkes R., Man E., Thind J., Yeung S., Joy A., Hoskins C. (2020). Biomater. Sci..

[cit124] Hennart S. L. A., Domingues M. C., Wildeboer W. J., Van Hee P., Meesters G. M. H. (2009). Powder Technol..

[cit125] Li M., Yaragudi N., Afolabi A., Dave R., Bilgili E. (2015). Chem. Eng. Sci..

[cit126] Chen Y. , Wang Y., Fleming J., Yu Y., Gu Y., Liu C., Fan L., Wang X., Cheng M., Bi L. and Liu Y., medRxiv, 2020, preprint, 10.1101/2020.03.10.20033795

[cit127] Fang E., Liu X., Li M., Zhang Z., Song L., Zhu B., Wu X., Liu J., Zhao D., Li Y. (2022). Signal Transduction Targeted Ther..

[cit128] Ball P. (2021). Nature.

[cit129] Johnston K. P., Maynard J. A., Truskett T. M., Borwankar A. U., Miller M. A., Wilson B. K., Dinin A. K., Khan T. A., Kaczorowski K. J. (2012). ACS Nano.

[cit130] Lu Y., Li Y., Wu W. (2016). Acta Pharm. Sin..

